# Magnetic resonance imaging for preoperative diagnosis in third molar surgery: a systematic review

**DOI:** 10.1007/s11282-022-00611-4

**Published:** 2022-04-09

**Authors:** Adib Al-Haj Husain, Bernd Stadlinger, Sebastian Winklhofer, Marco Piccirelli, Silvio Valdec

**Affiliations:** 1 Clinic of Cranio-Maxillofacial and Oral Surgery, Center of Dental Medicine, University of Zurich, Plattenstrasse 11, 8032 Zurich, Switzerland; 2 Department of Neuroradiology, Clinical Neuroscience Center, University Hospital Zurich, University of Zurich, Zurich, Switzerland; 3 Division of Periodontology, Department of Stomatology, Dental School, University of São Paulo, São Paulo, Brazil

**Keywords:** (MeSH): Magnetic resonance imaging, Third molar surgery, Oral surgery, Oral radiology, Inferior alveolar nerve, Systematic review

## Abstract

In recent years, magnetic resonance imaging (MRI) has made great strides through various technical improvements and new sequences, which have made it one of the most promising and leading imaging techniques in the head and neck region. As modern imaging techniques in dentistry aim to reduce radiation exposure, this systematic review evaluated the possibilities, advantages, and disadvantages of advanced imaging diagnostics using dental MRI and its evidence for clinical indications and limitations relevant to mandibular third molar (MTM) surgery. Two reviewers performed multiple database searches (PubMed MEDLINE, EMBASE, Biosis, and Cochrane databases) following the PICOS search strategy using medical subject headings (MeSH) terms, keywords, and their combinations. Ten studies were included in this systematic review. By providing high spatial resolution and excellent soft tissue contrast, black bone MRI sequences such as 3D Double Echo Steady State (DESS) and 3D Short Tau Inversion Recovery (STIR) imaging protocols have the potential to become a valuable alternative to cone-beam computed tomography (CBCT) in future dental clinical routines. Overall, radiation-free MRI represents another step toward personalized dentistry and improved decision-making that avoids ineffectiveness and minimizes risks in oral surgery by taking into account additional patient-side factors such as comorbidity, anatomical norm variations, and imaging biomarkers.

## Introduction

The extraction of partially erupted or impacted third molars is one of the most frequent oral surgical procedures in dental practice. Thanks to time-efficient, minimally invasive approaches aiming for hard tissue preservation and also novel techniques such as piezosurgery or laser therapy, surgical extraction of third molars has become a routine medical treatment that commonly proceeds without complications [1, 2]. However, due to the close positional relationship of the mandibular third molar (MTM) to the peripheral extracranial branches of the mandibular nerve, in particular the inferior alveolar nerve (IAN) and the lingual nerve (LN), advanced preoperative radiological assessment is of fundamental value for proper surgical case management, assessment of potential perioperative risks, and prevention of unpleasant postoperative complications.

Regarding the occurrence of transient iatrogenic IAN and LN damage associated with MTM surgery, there are heterogeneous datasets in the literature, estimating IAN injuries at approximately 4% (0.4–8.4%) [3–5] and LN injuries at 0.01–2% [6]. In this context, specific radiographic risk factors such as a close positional relationship between the MTM and the mandibular canal (MC), diversion of the MC, darkening of the MTM roots, and incomplete integrity of the osseous boundaries of the MC may indicate an increased risk for IAN injury [7–9]. Fortunately, in most cases, spontaneous healing occurs within the first 3–6 months after surgery [10]. However, if no signs of improvement are observed within this period, the risk of permanent neurosensory dysfunction increases, potentially resulting in a relevant compromise of the quality of life of the affected patients [11].

A conventional two-dimensional panoramic radiograph (PAN) is obtained in the daily clinical routine prior to MTM surgery. This overview image captures the entire oral cavity in a single image with relatively short radiation time and exposure (4–30 μSv) and provides anatomical information about the angulation and position of the MTM and the relative position of the MTM roots to the MC. In cases showing the aforementioned radiographic signs of risk, an overlap of anatomic structures, or displacement of the MC, two-dimensional imaging is not sufficient, and three-dimensional radiographic imaging modalities such as computed tomography (CT) or cone-beam computed tomography (CBCT) is indicated [12]. Despite the standardized grayscale value and insufficient soft tissue resolution, CBCT is considered the imaging modality of choice in dentomaxillofacial radiology due to greater accessibility, short examination time, lower radiation dose, and lower cost compared to conventional CT [13]. CBCT generates three-dimensional cross-sectional images of the examined craniofacial and dental structures with high spatial resolution at a radiation dose of approximately 18–200 µS and provides accurate information about relevant anatomical structures, such as the number and shape of MTM roots, the buccolingual location of the MC, and its cortical integrity [14]. However, generating this additional information, which may be elementary for the surgeon performing a high-risk MTM procedure, is associated with an increased lifetime risk for radiation-induced cancers given the increase in CBCT usage in various dental specialties [15, 16]. Considering the efforts to minimize or even eliminate radiation exposure in medical imaging according to the As Low As Reasonably Achievable (ALARA) principle and the proposed paradigm shift towards the As Low As Diagnostically Acceptable (ALADA) principle [17], several promising modalities such as low-dose CBCT or magnetic resonance imaging (MRI) have recently been integrated into dental imaging workflows, resulting in improved virtual surgical planning in oral and maxillofacial surgery with predictable intraoperative outcomes. Reports in the literature have shown that low-dose CBCT imaging protocols can provide confidential diagnostic information with an improved benefit–risk ratio compared to standard-dose CBCT in various clinical situations, e.g., implantology and detection of bony lesions in the jaw [18–20]. However, given the large number of manufacturer-specific scan parameters, modification and especially standardization of low-dose imaging protocols are still subject to research.

MRI is a non-invasive imaging technique that is one of the most promising and leading imaging modalities for the diagnosis of diseases and other conditions in the head and neck region [21]. A major advantage of MRI over conventional X-ray imaging is the high soft tissue contrast, which allows much better visualization of specific anatomical structures (e.g., nerves, blood vessels) using magnetic fields without exposing patients to ionizing radiation [22]. Despite the limitations in hard tissue imaging, MRI has advanced rapidly over the past two decades with various technical innovations and advanced imaging protocols, offering a wide range of new diagnostic capabilities in dentistry [23]. The challenges in visualizing dentition with MRI are due to the dense organic constitution, only allowing a limited molecular movement of hydrogen nuclei. This is further affected by the rapid signal decay after radiofrequency excitation so that the transverse relaxation time (T2*) of more than 1 ms, which is necessary to digitize MRI signals for visualization of mineralized dental tissue, cannot be obtained [24, 25]. Recently introduced “black bone” MRI sequences such as 3D double-echo steady-state (DESS) and 3D short tau inversion recovery (STIR) have contributed significantly to an optimized simultaneous visualization of soft and hard tissue, allowing the visualization of the IAN within the MC near the MTM roots without significant diagnostic limitations compared to conventional CBCT [26, 27]. In addition, continuous visualization of the IAN and LN and their finest ramifications are possible [28, 29], improving the diagnostic capabilities in the third molar region and positively impacting preoperative planning in MTM surgery [30, 31]. The residual limitations of MRI in the oral cavity are susceptibility to motion artifacts, complex anatomic courses of small-sized blood vessels and nerves, and image distortion and artifacts due to magnetic field inhomogeneities caused by metallic dental restorations [32].

However, the growing number of MRI studies in dentistry underlines the relevance and the perspectives opened by the targeted use of specific instruments such as mandibular dental coils [33], which represent a tremendous resource for imaging the mandibular third molar region in the oral cavity. Nevertheless, evidence-based guidelines for case-specific indications and limitations are still lacking. Therefore, the aim of this systematic review was to evaluate the indications and limitations of different MRI protocols in terms of diagnostic quality and potential benefits in MTM surgery and to provide guidelines based on daily clinical questions by examining the following PICO question: does preoperative MRI provide accurate and feasible preoperative diagnostic information in healthy volunteers and patients undergoing MTM surgery?

## Materials and methods

### Search strategy, information sources, and eligibility criteria

This systematic review was performed in accordance with the preferred reporting items for systematic reviews and meta-analysis (PRISMA) guidelines. A search for articles on the use of dental MRI in MTM surgery was performed using the Pubmed MEDLINE, EMBASE, BIOSIS, and Cochrane electronic databases. All English and German articles from 1989 until December 2021 were considered. Data searches were performed using the following MeSH terms, keywords, and their combinations with Boolean operators: third molar extraction, magnetic resonance imaging, nuclear magnetic resonance imaging, diffusion tensor imaging, ultrashort echo-time, neurography, visualization, inferior alveolar nerve, lingual nerve, and mandibular nerve. Thereby the following PICO question was assessed: P-population: human studies with participants undergoing MRI prior to MTM surgery, or healthy patients undergoing MRI to assess the anatomical and spatial relationship of the MTMs in the third molar region; I-intervention: magnetic resonance imaging; C-control: not applicable; O-outcome: feasibility and accuracy of preoperative radiological assessment of the mandibular third molar region; and S-study designs: clinical trials (Table 1). This systematic review was not registered in the International Prospective Register of Systematic Reviews (PROSPERO) platform (no protocol number available).

**Table 1:** Search strategy according to the focused question (PICO)

Focused question (PICO)	Does preoperative MRI provide accurate and feasible diagnostic information in healthy volunteers and patients undergoing MTM surgery?	
Search strategy	Population	Human studies (patients and/or healthy subjects), aged older than 12 years undergoing MRI prior to MTM surgery #1—((third molar extraction [MeSH]) OR (inferior alveolar nerve [MeSH]) OR (lingual nerve [MeSH]) OR (mandibular nerve [MeSH]) OR (trigeminal nerve [MeSH]))
	Intervention	Magnetic resonance imaging #2—((magnetic resonance imaging [MeSH]) OR (MRI) OR (nuclear magnetic resonance imaging [MeSH]) OR (NMR) OR (diffusion tensor imaging [MeSH]) OR (DTI) OR (ultra-short echo-time [MeSH]) OR (UTE) OR (maxillofacial imaging)) #3—((visualization) OR (neurography))
	Comparison	Conventional preoperative radiological assessment #4—((computed tomography [MeSH]) OR (cone-beam computed tomography [MeSH]) #5—(panoramic radiography [MeSH])
	Outcome	Feasibility and accuracy of preoperative radiological assessment of MTM region using MRI #6—((accuracy) OR (feasibility) OR (signal-to-noise-ratio [MeSH]))
	Search combination(s)	(#1) AND (#2 or #3) OR (#6)

Studies were included based on the following criteria: (1), human studies with participants undergoing MRI prior to MTM surgery, or healthy patients undergoing MRI to assess the anatomical and spatial relationship of the MTMs in the third molar region in randomized or nonrandomized controlled trials and cohort studies; (2), study participants older than 12 years; (3), availability of the full text; and (4), articles in English or German language. Exclusion criteria were: (1), animal, cadaveric, in vitro studies, systematic reviews, narrative reviews, and case reports; (2) patients with other additional pathologies requiring MRI examination of the region of interest.

### Data extraction and collection

**Fig. 1:**
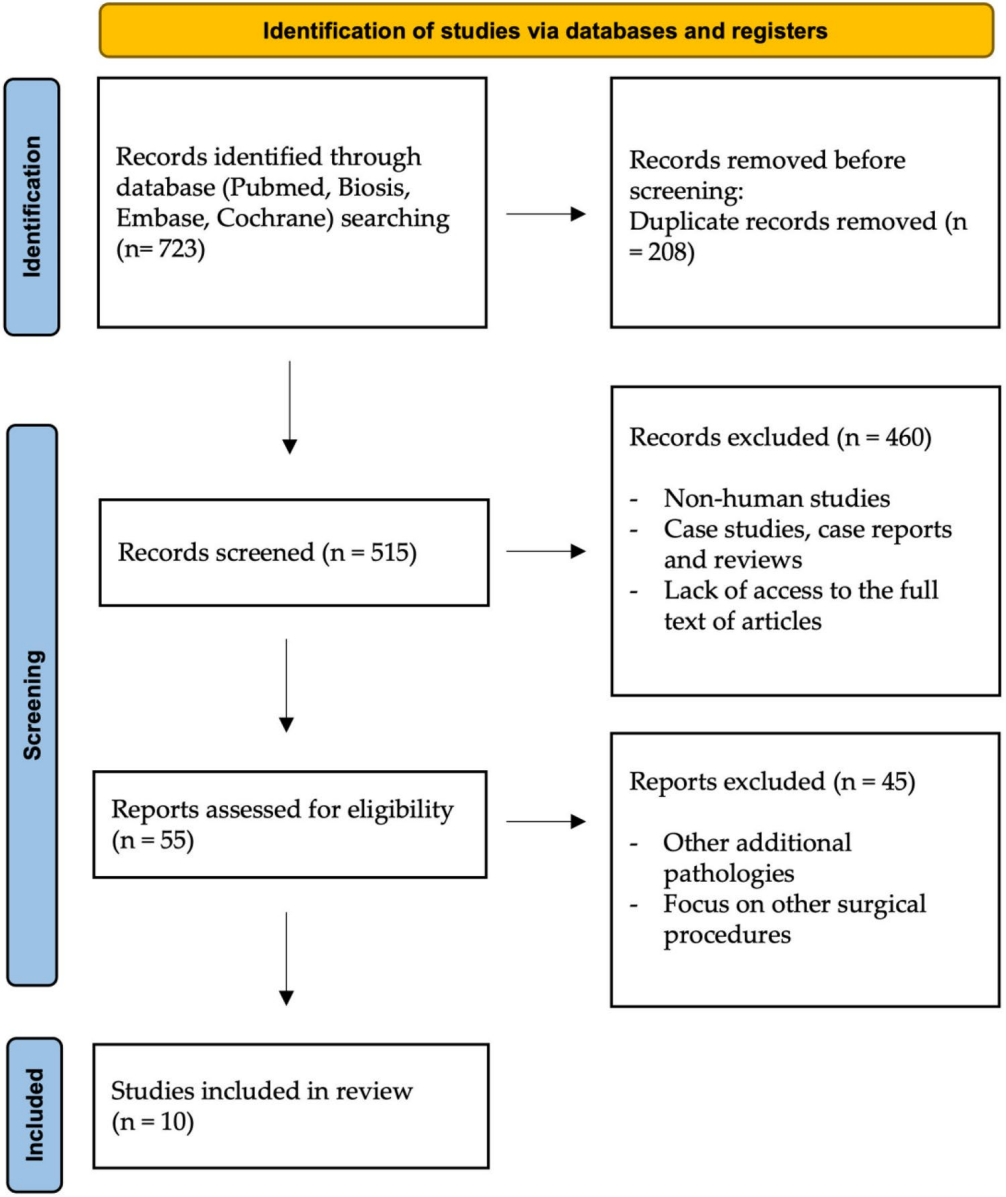
PRISMA flow diagram showing the article selection in this review

Two reviewers (A.A.H. and S.V.) conducted the literature searches independently to minimize potential reviewer bias. From all databases, the data were transferred to EndNote (Clarivate, Sydney, Australia) and all duplicates were removed. First, titles and abstracts were screened, and then 55 studies were selected for full-text analysis. If ambiguities occurred, they were clarified through discussion. Finally, ten articles within the scope of this review examining the use of magnetic resonance imaging in MTM surgery were evaluated (Fig. 1).

For each study, the following parameters were recorded by two reviewers (A.A.H and S.V. separately): general information (study design, author, year, country, number of patients treated, age range of patients treated), MRI imaging protocol parameters (MR device, MRI sequence, field strength, acquisition time, type of MRI coil), and outcomes (feasibility and accuracy).

### Quality assessment in individual studies

The methodology of the included studies for risk of bias was evaluated based on a modified short version of the Strengthening the Reporting of Observational Studies in Epidemiology (STROBE) according to Edwards et al. [34]. The risk of bias was assessed using 18 criteria from the STROBE statement, whereby a total score of 15 or more out of 18 was considered low risk of bias, a score of 11–14 as medium risk of bias, and studies with a score of 10 or less as high risk of bias.

## Results

### Study selection

In accordance with the purpose of this systematic review, the literature search identified 723 potentially relevant studies. After duplicates were removed, 515 articles remained. In the first step of study selection, the titles and abstracts were checked, and then 460 articles were excluded (non-human studies, case studies, case reports and reviews, lack of access to the full text of articles). The remaining 55 articles were subjected to full-text screening in a second step. In this process, 45 articles were excluded because they did not meet the inclusion criteria. Finally, ten articles were within the scope of this review.

### Study characteristics

In the ten studies included in this systematic review (Miloro et al. [42]; Ferretti et al. [55]; Chau et al. [43]; Kirnbauer et al. [45]; Kang et al. [56]; Burian et al. [30]; Beck et al. [44], Al-Haj Husain et al. [31]; Al-Haj Husain et al. [28]; Valdec et al. [26]), 202 patients underwent an MRI scan preoperatively between 1997 and 2021 at MR field strengths of 1, 1.5, or 3 Tesla using various MRI sequences. The included patients’ age ranged from 12 to 65 years with an acquisition time of 3:30 min to about 20 min (Table 2).

**Table 2:** Characteristics of included studies

Study number	Author, year, country	Study	Sample size	Age	Study objectives	MRI sequences	Field strengths	Type of MRI coil	Acquisition time	MR device	Outcome (feasibility/accuracy)
1	Miloro et al. 1997, USA	Assessment of the lingual nerve in the third molar region using magnetic resonance imaging	10	21–35 years	Determination of the precise in situ location of the lingual nerve in the third molar region by high-resolution MRI	Phase encode time reduction acquisition (PETRA) sequence	1.5 T	surface coil	9:17 min	Signa MRI Unit, General Electric Medical Systems, Milwaukee, Wisconsin, USA	Precise lingual nerve visualization in the third molar region using the PETRA sequence. Assessment of the nerve diameter, the different shapes of the nerve, and the mean vertical and horizontal distances to the lingual crest and the lingual plate of the mandible
2	Ferretti et al. 2009, Italy	Dental magnetic resonance imaging: study of impacted mandibular third molars	29	15–28 years	Evaluation of the relationship between the mandibular canal and impacted mandibular third molars using MRI	T1 weighted (short repetition time (TR)/short echo time (TE)) with spin echo (SE) technique or proton-density (PD)-weighted (long TR/short TE) with turbo spin echo (TSE) technique	1.0 T	radiofrequency head coil	N/A	Magnetom Expert; Siemens Medical Systems, Erlangen, Germany	MRI provided a good depiction of anatomical details of the mandibular canal and its relationship to the MTMs
3	Chau et al. 2012, Hong Kong	Comparison between the use of magnetic resonance imaging and cone-beam computed tomography for mandibular nerve identification	11	N/A	Evaluation of the reliability of the radiographic assessment of the mandibular nerve in MRI or the mandibular canal in CBCT prior to mandibular third molar extraction	T1 weighted Volumetric Interpolated Breath-hold Examination (VIBE) sequence with fat suppression	3.0 T	12-channel birdcage head and neck coil	4 min	Magnetom Trio Tim syngo, Siemens Medical Solutions, Erlangen, Germany	MRI provided better results in identifying the inferior alveolar nerve/canal and can be used to identify the mandibular nerve in cases that are not accurately visualized on CBCT
4	Kirnbauer et al. 2018, Austria	Assessment of impacted and partially impacted lower third molars with panoramic radiography compared to magnetic resonance imaging—a proof of principle study	28	13–24 years	Comparison of panoramic radiography and MRI in preoperative radiological assessment considering the positional relationship of impacted and partially impacted mandibular thirds	3D turbo spin echo (TSE) sequence and 3D constructive interference in steady-state (CISS) sequence	3.0 T	8-channel receive-only CPC coil	20:31 min	Magnetom Trio, a TIM System, Siemens AG, Erlangen, Germany	MRI provided the same information compared with panoramic radiography, with the added advantage of providing three-dimensional information generated without radiation
5	Kang et al. 2018, China	Investigation of Zero TE MR in preoperative planning in dentistry	22	26–65 years	Comparison of preoperative assessment of the positional relationship between the mandibular third molar and the mandibular canal in mandibular third molar surgery using CBCT and zero echo-time MRI	Ultrashort and zero echo-time (ZTE) MRI	3.0 T	8-channel head neck coil	3:30 min	MR Discovery 750 W, GE Healthcare, Milwaukee, Wisconsin, USA	ZTE MRI demonstrated the delineation of the teeth and mandible and provided superior visualization of the mandibular canal compared to CBCT Identical results were obtained in the assessment of the spatial relationship between the mandibular third molar and the mandibular canal
6	Burian et al. 2020, Germany	MRI of the inferior alveolar nerve and lingual nerve—anatomical variation and morphometric benchmark values of nerve diameters in healthy subjects	30	21–32 years	Imaging of the inferior alveolar nerve and lingual nerve in the mandibular third molar region using black bone MRI sequences	3D Short tau inversion recovery (STIR), 3D Double-echo steady-state (DESS), and 3D T1 fast field echo (FFE)	3.0 T	16-channel Head and Neck Spine Coil	s 5:31 min	Ingenia Elition, Philips Healthcare, Best, the Netherlands	Possibility of accurate visualization of the inferior alveolar nerve and the lingual nerve in the mandibular third molar region. STIR provided the best signal-to-noise ratio. Accurate discrimination of the tissue composition of the mandibular neurovascular bundle was achieved
7	Beck et al. 2021, Austria	Is MRI a viable alternative to CT/CBCT to identify the course of the inferior alveolar nerve in relation to the roots of the third molars?	53	N/A	Assessment of the spatial relationship between the inferior alveolar and the mandibular third molar using MRI or CT/CBCT images	PD T2 TSE fat saturation (FS) axial, PD TSE FS coronal	3.0 T	64 channel head-and-neck coil	10:44 min	Magnetom Skyra, Siemens Healthcare, Erlangen, Germany	Good inter- and intra-rater agreement in assessing the spatial relationship between IAN and MTM. Additionally, MRI offered advantages in identifying of accessory IAN in comparison to CT/CBCT
8	Al-Haj Husain et al. 2021, Switzerland	Mandibular third molar surgery: intraosseous localization of the inferior alveolar nerve using 3D double-echo steady-state MRI (3D-DESS)	19	18–63 years	Evaluation of the intraosseous position of the inferior alveolar nerve within the mandibular canal’s osseous boundaries prior to third molar extraction treatment using preoperative cone-beam computed tomography and magnetic resonance imaging. Assessment of a conversion factor between both imaging modalities	3D-DESS with water excitation sequence	3.0 T	64 channel head-and-neck coil	12:24 min	Skyra (release VE11c), Siemens Healthi- neers, Erlangen, Germany	Accurate simultaneous visualization of the nerve tissues within osseous boundaries. A conversion factor from IAC in CBCT and MRI to IAN in MRI was determined
9	Al-Haj Husain et al. 2021, Switzerland	Preoperative visualization of the lingual nerve by 3D double-echo steady-state MRI in surgical third molar extraction treatment	19	18–63 years	Evaluation of the focal and continuous anatomy of the lingual nerve in the surgically relevant region before mandibular third molar surgery	3D-DESS with water excitation sequence	3.0 T	64 channel head-and-neck coil	12:24 min	Skyra (release VE11c), Siemens Healthi- neers, Erlangen, Germany	Preoperative clarification of the focal and continuous anatomy of the lingual nerve in the third molar region with high reliability
10	Valdec et al. 2021, Switzerland	Comparison of Preoperative Cone-Beam Computed Tomography and 3D-Double Echo Steady-State MRI in Third Molar Surgery	19	18–63 years	Evaluation of the reliability of assessing the positional relationship between the inferior alveolar nerve and the mandibular third molar using CBCT, MRI, and CBCT/MRI image fusion	3D-DESS with water excitation sequence	3.0 T	64 channel head-and-neck coil	12:24 min	Skyra (release VE11c), Siemens Healthi- neers, Erlangen, Germany	Moderate intra- and inter-rater agreement for the positional relationship was observed, independent of the reader’s experience. MRI provided superior diagnostic benefit regarding early detection of inflammation

### Quality assessment in individual studies

The risk of bias assessment in each study was low in 7 and medium in 3 studies. An overview of the percentage responses for each topic is displayed in Table 3.

**Table 3:** Quality assessment of included studies

Checklist	Miloro et al. [42]	Ferretti et al. [55]	Chau et al. [43]	Kirnbauer et al. [45]	Kang et al. [56]	Burian et al. [30]	Beck et al. [44]	Al-Haj Husain et al. [31]	Al-Haj Husain et al. [28]	Valdec et al. [26]
*Objectives: *clearly formatted	+	+	+	+	+	+	+	+	+	+
*Study design:* described in detail	+	+	+	+	+	+	+	+	+	+
*Settings:* described in terms of location; and relevant dates	+ ; +	+ ; +	+ ; +	+ ; +	+ ; +	+ ; +	+ ; +	+ ; +	+ ; +	+ ; +
*Participants:*eligibility criteria; and methods of selection described	+ ; +	+ ; +	−; +	+ ; +	+ ; +	+ ; +	+ ; +	+ ; +	+ ; +	+ ; +
*Bias:* any efforts to address potential sources of bias described	+	−	+	+	+	+	+	+	+	+
*Sample size:* explanation of derivation; adequate	−; +	−; +	−; +	−; +	−; +	−; +	+ ; +	−; +	−; +	+ ; +
*Statistical Methods:* described; appropriate for data	−; +	−; +	+ ; +	+ ; +	+ ; +	+ ; +	+ ; +	+ ; +	+ ; +	+ ; +
*Participants:* described	+	+	+	+	+	+	+	+	+	+
*Outcome data:* number of outcome events reported	+	+	+	+	+	+	+	+	+	+
*Other analysis:* any other analyses conducted reported	−	+	−	−	−	+	+	+	+	+
*Limitations:* limitations of the study; and any potential bias discussed	−; +	−; -	−; +	+ ; +	+ ; +	+ ; +	+ ; +	+ ; +	+ ; +	+ ; +
*Interpretation:* overall interpretation of results provided	+	+	+	+	+	+	+	+	+	+
*External validity:* generalizability of the results discussed	+	−	+	−	−	−	−	−	+	−
Total out of 18 (percentage)	14 (78%)	12 (67%)	14 (78%)	15 (83%)	15 (83%)	16 (89%)	17 (94%)	16 (89%)	17 (94%)	17 (94%)

## Discussion

### Magnetic resonance imaging—overview

Dental MRI is a modern, non-invasive cross-sectional imaging technique that provides three-dimensional, detailed anatomical images of the oral cavity and its contents. High-resolution MR images are generated by combining magnetic fields of different strengths, magnetic field gradients, high-frequency magnetic pulses exciting hydrogen protons and magnetic properties of living tissue. Since living tissue in humans is composed of about 70% water, this property is exploited to produce accurate MR images of soft tissues such as internal organs, tendons, ligaments, or muscles. Consequently, in contrast to conventional X-ray, the various image representations in MRI do not reflect the general tissue density but the proton density of the individual tissues [35, 36].

Continuous improvement and optimization of resolution and image quality are based on modifying all three fundamental factors for biomedical imaging—the patient, the imaging device, and the image detector. In this context, the signal-to-noise ratio, which evaluates the ratio between the desired signal intensity and the background noise, and the spatial resolution, which depends on the image voxel size, are the most fundamental parameters for MR image quality [37]. Signal-to-noise ratio can be optimized by increasing the voxel size, the field of view, time to repeat, the slice thickness, and the number of signal acquisitions or decreasing the matrix size and bandwidth by applying specific coils [38]. However, it should be considered that image definition decreases as the signal-to-noise ratio increases. A mandibular dental coil is used to reduce the field of view and increase image resolution without decreasing the signal-to-noise ratio [35]. Depending on the medical issue, especially in oncological imaging or the detection of inflammation, a contrast agent containing gadolinium may be administered intravenously to increase the informative value of MRI. Various changes and advancements in MRI and the introduction of new MRI sequences have steadily improved diagnostic capabilities in recent years, allowing the determination of quantitative parameters such as tissue perfusion, oxygen concentration, and diffusion.

However, it should be noted that there are potential contraindications to MR examinations which include electronic implants, intracorporeal pacemakers, brain and spinal cord stimulators, insulin pumps, cochlear implants, or metallic foreign bodies in soft tissue, severe anaphylactic reactions to MR contrast agents, claustrophobia, and pregnancy. Nowadays, the concept of absolute or relative contraindications has been largely abandoned. Instead, the individual patient situation should be taken into account, including the scan indication, the exact implantation clarification and an individual risk assessment of the examination. Many points that were previously considered as absolute contraindications are now potentially suitable or conditional for MR after clarification and after adjustment of the scan parameters.

### Magnetic resonance imaging in MTM surgery

The analysis focused on the impact of imaging protocols and device-specific parameters on the accuracy and feasibility of visualization of the third molar region in MTM surgery performed according to modern surgical concepts. In this context, factors influencing the MR images, such as the MR device, the effect of field strength, type of MRI coil, acquisition time, and MRI sequence on MR images were evaluated. Considering the continuously increasing number of MRI studies in dentistry and the related wide range of applications in preoperative diagnostics, it is necessary to evaluate the evidence of added benefit as well as the indications and limitations of the individual MRI protocols and to make suggestions for optimized individual clinical decision-making in MTM surgery.

The conventional X-ray-based imaging techniques commonly used in MTM surgery, such as PAN or three-dimensional CBCT, can visualize various pathologies and the surgically relevant structures, such as the MTM, the radiolucent MC with the surrounding osseous boundaries, and maxillary sinuses. However, the increasing use of CBCT in MTM surgery is suggested to lead to an additional increase in radiation-induced cancer incidence by 0.46 [39], which is particularly relevant to repeated radiation exposure in radiosensitive, genetically susceptible young adolescents [15]. Other studies suggest that diagnostic radiation exposure from dental radiography may be associated with an increased risk of thyroid cancer and meningioma [40]. Therefore, radiation-free MRI presents a valid alternative that can be used for medical diagnosis, monitoring of treatment, and follow-up care.

Modern surgical concepts in MTM surgery aim for low-risk, minimally invasive, hard tissue preserving approaches, whereby a multimodal approach considering the medical history, intraoperative findings and preoperative radiographic evaluation of the third molar region is crucial to minimize perioperative risks [41]. The depth, angulation and orientation of impaction, number of roots and their morphology, proximity to the IAN and LN and the presence of other pathological processes should always be considered [41]. The results of this systematic review confirm the feasibility and accuracy of preoperative visualization of the MTM region by MRI, with a special focus on the most vulnerable structures, e.g., the IAN and LN. The first MRI studies addressing this issue in MTM surgery were conducted in the late 1990s, whereby phase encode time reduction acquisition (PETRA) sequence, an imaging protocol developed specifically for this purpose, was used to assess the LN visualization and various surgically relevant quantitative and qualitative parameters [42]. The older MRI studies were performed with a field strength of 1 Tesla and the use of conventional, nonspecific MR sequences, resulting in a low signal-to-noise ratio what could be considered insufficient and a limitation from today's perspective. However, it was possible to visualize the neurovascular bundle in the mandibular third molar region as a moderately hyperintense signal, resulting in good contrast due to the lower signal from the bony boundaries of the MC, without distinguishing the nerve tissue from blood vessels.

Current developments are moving toward novel MRI protocols suitable for dental imaging. Special "black bone" MRI sequences such as 3D double-echo steady-state (DESS) and 3D short tau inversion recovery (STIR) sequences provided high-resolution and high-contrast images that allow simultaneous visualization of the inferior alveolar nerve tissue within the osseous boundaries of the mandibular canal, providing a reliable assessment of the positional relationship of MTMs [26, 28, 30, 31]. In these dedicated MRI protocols using the water excitation/fat suppression technique, the IAN and LN appear as a highly hyperintense signal and can be distinguished from the MC due to the myelin layer surrounding the nerves [29].

**Fig. 2:**
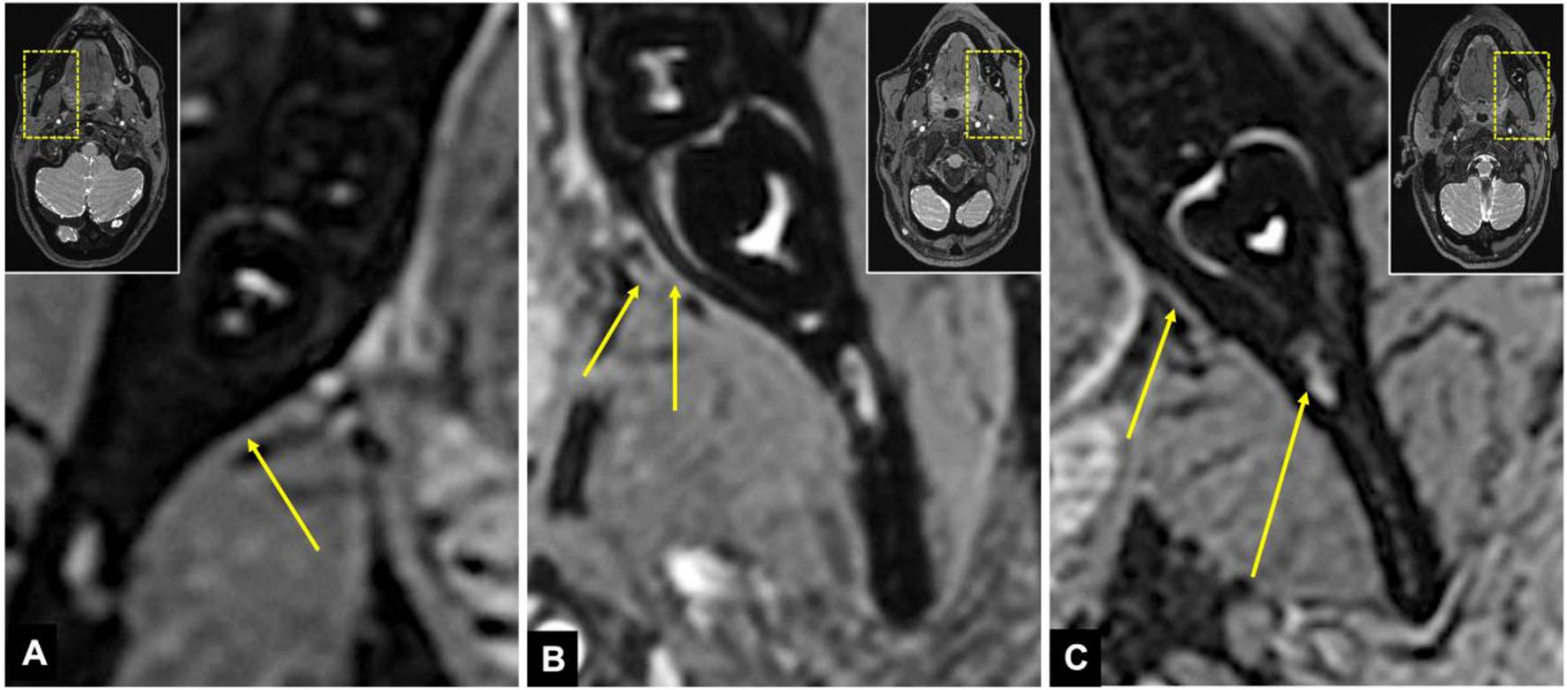
Visualization of the lingual nerve in the mandibular third molar region in axial 3D double-echo steady-state (3D-DESS) MRI reconstructions: **a** The arrow indicates the hyperintense signal intensity of the lingual nerve as it enters the mandibular third molar region; **b** The shorter arrow visualizes the main branch of the lingual nerve in the mandibular third molar region, while the longer arrow represents the branch described in the literature as the "gingival branch of the lingual nerve" or "collateral nerve twig"; **c** another example where the shorter arrow represents the lingual nerve while the longer arrow visualizes the inferior alveolar nerve

The 3D DESS sequence allowed focal and continuous visualization of the LN from the foramen ovale to the MTM region with high reproducibility [28] (Fig. 2). This preoperative diagnosis of the exact location of the LN could reduce complications in complex angulated, deeply impacted MTMs and other dentoalveolar surgical procedures performed in anatomic proximity to the LN. In addition, this MRI protocol is considered superior to other black bone MRI protocols in terms of radiographic assessment of quantitative parameters of the LN [28, 30].

**Fig. 3:**
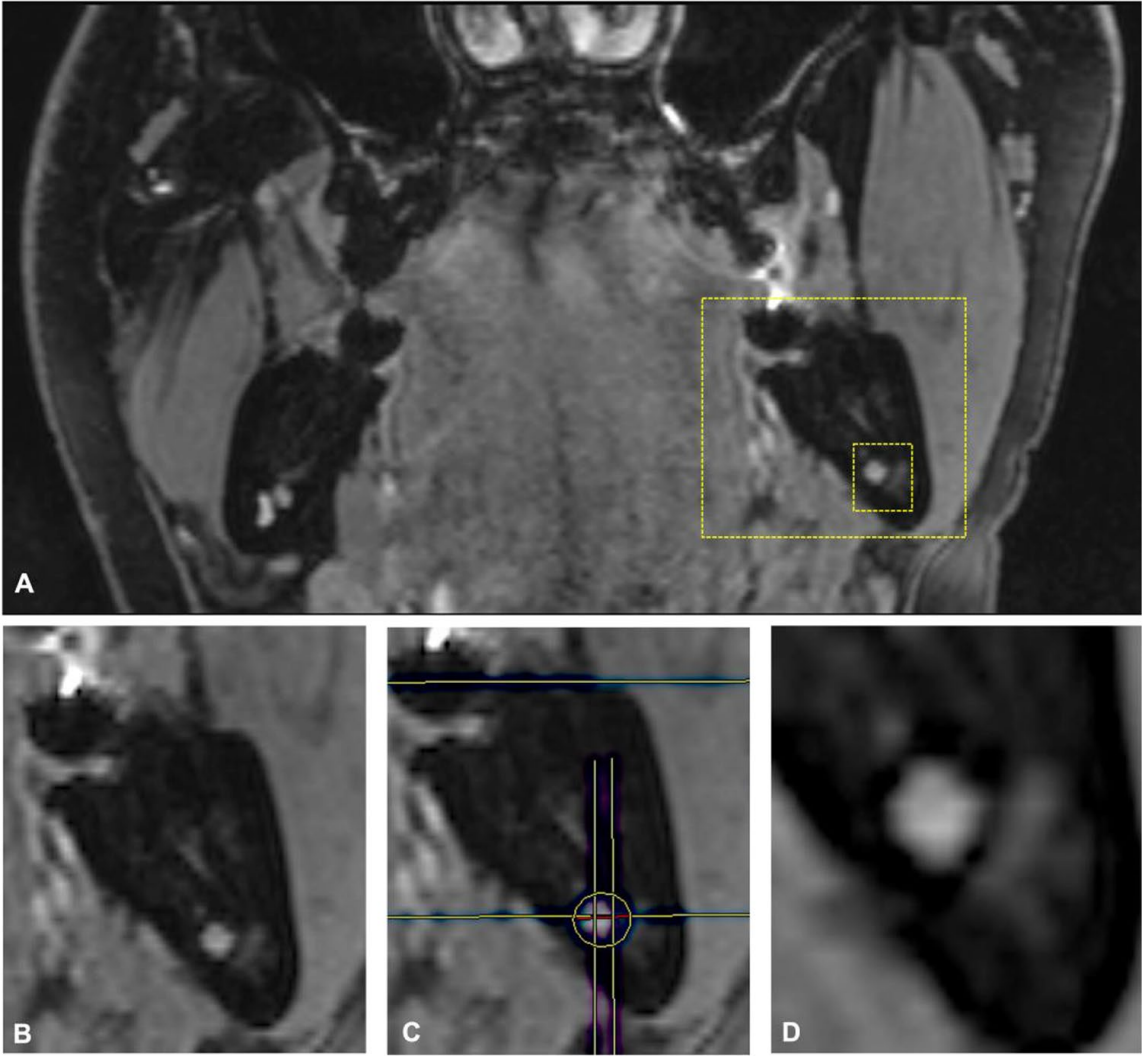
The evaluation of the intraosseous position of the inferior alveolar nerve based on the coronal 3D double-echo steady-state (3D-DESS) MRI reconstructions is visualized: **a** overview in the coronal reference layer; **b** magnification of the situation in the mandibular third molar region; **c** subdivision of the mandibular canal into six segments and check for the presence of MRI signal hyperintensities; **d** visualization of buccal displacement of the inferior alveolar nerve by the roots of the mandibular third molar in the segments proximal to the contact site. This phenomenon is also frequently described as the "snake phenomenon" in the literature

However, Burian et al. reconfirmed that, as of today, black bone MRI sequences are best suited for this medical question. The STIR imaging protocol was superior and provided the most promising signal-to-noise ratio and nerve–muscle contrast to noise ratio for the IAN and the LN [30]. Al-Haj Husain et al. demonstrated that the preoperative intraosseous localization of the IAN within the bony borders of the MC is possible and accurate. Thereby, the IAN showed the highest localization within the central MC segments. Additionally, the retention type of the MTM was revealed to influence the intraosseous localization of the IAN, with the nerve being displaced by the MTM in the segments proximal to the contact site [31] (Fig. 3). This phenomenon is described in the literature as the "snake phenomenon". This additional generated information could be of interest to the performing surgeon in high-risk MTM surgeries, leading to improved strategies for perioperative management and thus better outcomes.

**Fig. 4:**
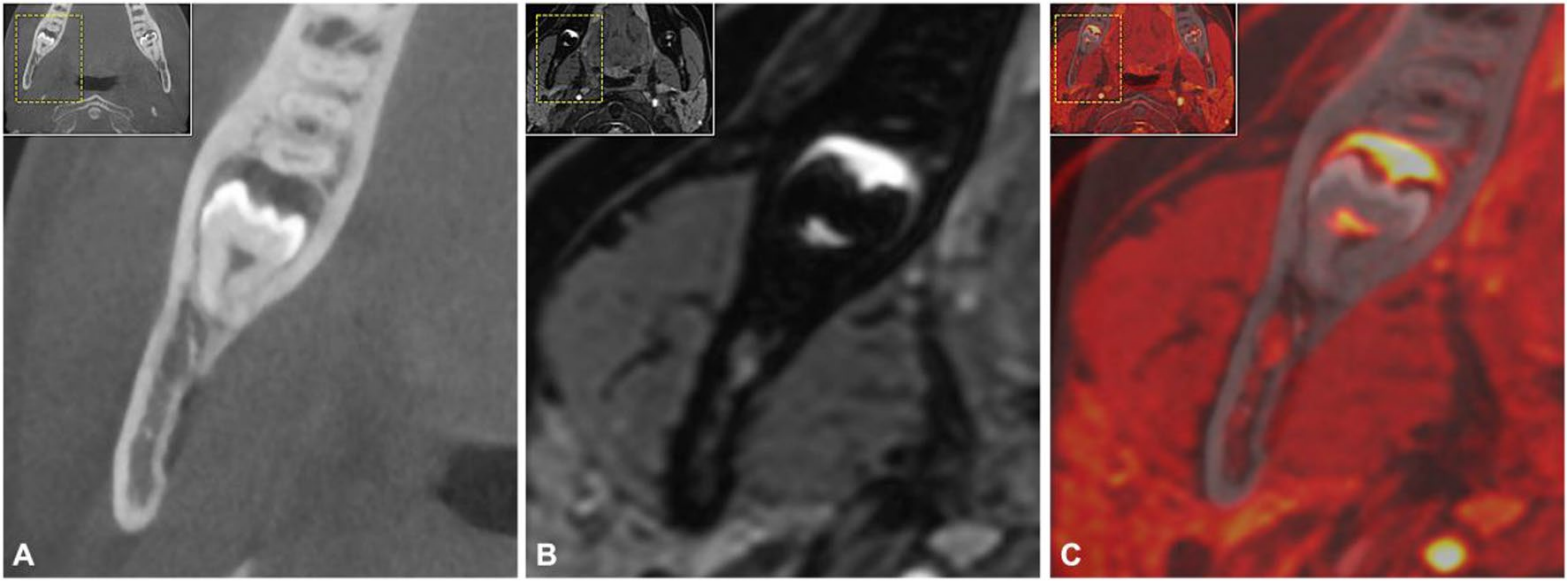
**a** CBCT, **b** MRI, and **c** fused CBCT/MRI imaging of the positional relationship between the mandibular canal, respectively, inferior alveolar nerve and the mandibular third molar. This figure displays the advantages and disadvantages of each imaging modality and their respective limitations. Depending on the imaging modality, relevant information is provided, such as direct visualization of the inferior alveolar nerve (**b**) or only the osseous boundaries of the MC (**a**). In addition, an inflammatory process most consistent with a follicular cyst is also visualized

In addition to depicting the anatomic course of the IAN and LN, the results obtained in this review demonstrated the utility of MRI in assessing the positional relationship between the IAN and MTM. Regarding the reliability of the assessment of the positional relationship, numerous reports comparing PAN or CBCT with MRI or fused CBCT-MRI images were evaluated. In general, MR imaging using T1 weighted volumetric interpolated breath-hold examination (VIBE) sequence with fat saturation [43], standard investigation MR protocols of the jaw region [44], or blackbone MRI sequences modified specifically for this purpose [26] offered similar results compared with CBCT and was even superior in cases where the MC could not be accurately visualized on CBCT. Compared to PAN, MRI using 3D turbo spin echo (TSE) and 3D constructive interference in steady-state (CISS) sequence provided the same information, with the added advantage of providing three-dimensional information of the region of interest without radiation exposure [45]. In general, it can be concluded that different MRI protocols of the included studies, with their respective advantages and disadvantages, are suitable for determining the relative position of the MTM to the IAN and provide superior visualization of the mandibular canal compared with CBCT. Using CBCT, it is only possible to indirectly visualize the location of the IAN via its cortical bone boundaries, which can be problematic as the MC is difficult or impossible to delineate in CBCT in approximately 20–40% of cases due to low or absent corticalization [46]. In CBCT, an apparent underestimation of the shape and volume of the neurovascular bundle of 1.5 to 5 mm thickness can be observed, thus the use of MRI can contribute significantly to a safer surgical approach [47]. In addition to the now qualitatively and quantitatively good and radiation-free visualization capabilities of bone tissue, the internal structure and microarchitecture of bone could also be assessed preoperatively, providing a new option for preoperative evaluation of the bone quality and allowing the selection of the most appropriate treatment option [48]. MRI scans also enable added diagnostic value compared to CBCT and CT due to their superior soft tissue contrast [26]. Thereby, oral mucosa or gingiva, neurovascular structures such as the IAN or LN in particular, or the dental pulp can be depicted directly [49, 50]. Compared with CBCT or MR imaging alone, the use of fused CBCT/MRI images could offer advantages in preoperative radiographic position assessment in borderline cases that are difficult to evaluate (Fig. 4). This might be especially relevant for borderline cases in which CBCT did not show the continuous osseous boundaries of the MC, while MRI nevertheless revealed a non-contact situation between the roots of the MTM and the IAN [26].

Various modifications and advancements of MRI, such as the introduction of functional MRI (fMRI) and the combination with positron emission tomography (PET), and the implementation of new MRI sequences, have steadily improved the diagnostic capabilities. The drive towards using 3 Tesla MRI is fueled by the advantages of improved signal-to-noise ratio, contrast-to-noise ratio, and high image resolution for dental applications. In many cases, these advantages will allow higher spatial resolution than lower field strength MRI. Alternatively, 1.5 Tesla MRI devices can be used with mandibular dental coils [51] or intraoral coils [52], optimizing image quality and shortening acquisition times. The previously very long scan times of up to 30 min and the low image quality with insufficient resolution, which were considered unsuitable for clinical routine, could, therefore, be overcome and currently be reduced to the range of about 3 min [53]. Furthermore, initial efforts are underway to offer out-of-hospital MRI examinations using recently developed bedside MRI scanners and additional specific equipment, which is an attractive option to make dental MRI scans more time and cost efficient. Nevertheless, further enhancements regarding standardization are still needed to enable the targeted use of MRI scans in routine oral surgical procedures. Considering the artifacts in MRI, motion artifacts, field inhomogeneity, and artifact-causing metallic dental restorations are the major challenges in MR imaging of the oral cavity. It must be taken into account that radiation-based techniques such as CBCT or CT can also cause pronounced artifacts. However, specific applications for artifact suppression are already established and should be further optimized [49, 54].

According to the results of this systematic review, various MRI protocols were suitable for preoperative imaging of the MTM region. However, the articles cited in this review show that MRI-based radiological assessment achieves comparable results to CBCT-based surgical planning without ionizing radiation. The additional information obtained may positively impact various preoperative planning in oral surgery and allow better prediction of surgical difficulties before surgery, leading to a safer surgical approach and a reduction in postoperative discomfort due to nerve damage. Although there is much heterogeneity in the literature related to scanning parameters, MRI currently represents the only promising imaging modality that provides non-invasive direct imaging of the neurovascular bundle, capable of discriminating the neural tissue from the blood vessels. More studies, including randomized control trials, should be performed investigating the benefit of each MRI protocol in MTM surgery to provide an evidence-based understanding of their use and more information about their impact on clinical outcomes. With improved cost-effectiveness and considering the improved risk–benefit ratio, the use of black bone MRI sequences such as DESS and STIR can present a valid alternative that can be used for medical diagnosis, monitoring of treatment, and follow-up care.

## Conclusion

From a clinical perspective, the targeted use of dental MRI in indicated MTM sugical cases could support or even replace radiation-based CBCT imaging in preoperative radiological assessment. As shown by the results of these studies, the use of "black-bone" MRI sequences, which have recently been increasingly investigated and modified, provides highly confidential and reliable additional preoperative diagnostic information, improved soft tissue resolution, and high sensitivity in detecting pathologic changes. Consequently, dental MRI could provide the performing surgeon with beneficial information preoperatively, enabling improved personalized therapeutic approaches in high-risk MTM surgery.
